# Supplementary material for: Towards unified AI-driven fracture mechanics: the extended deep energy method (XDEM)
Source: Nat Commun. 2026 Aug 15;17:8492. doi: 10.1038/s41467-026-76748-1 (PMC13484471; doi:10.1038/s41467-026-76748-1)
Supplement: Supplementary file 3 [file 41467_2026_76748_MOESM3_ESM.zip › pic/Results/Stress_intensity_factor/Mixed/beta_75/crack_sigma22_beta75.pdf]

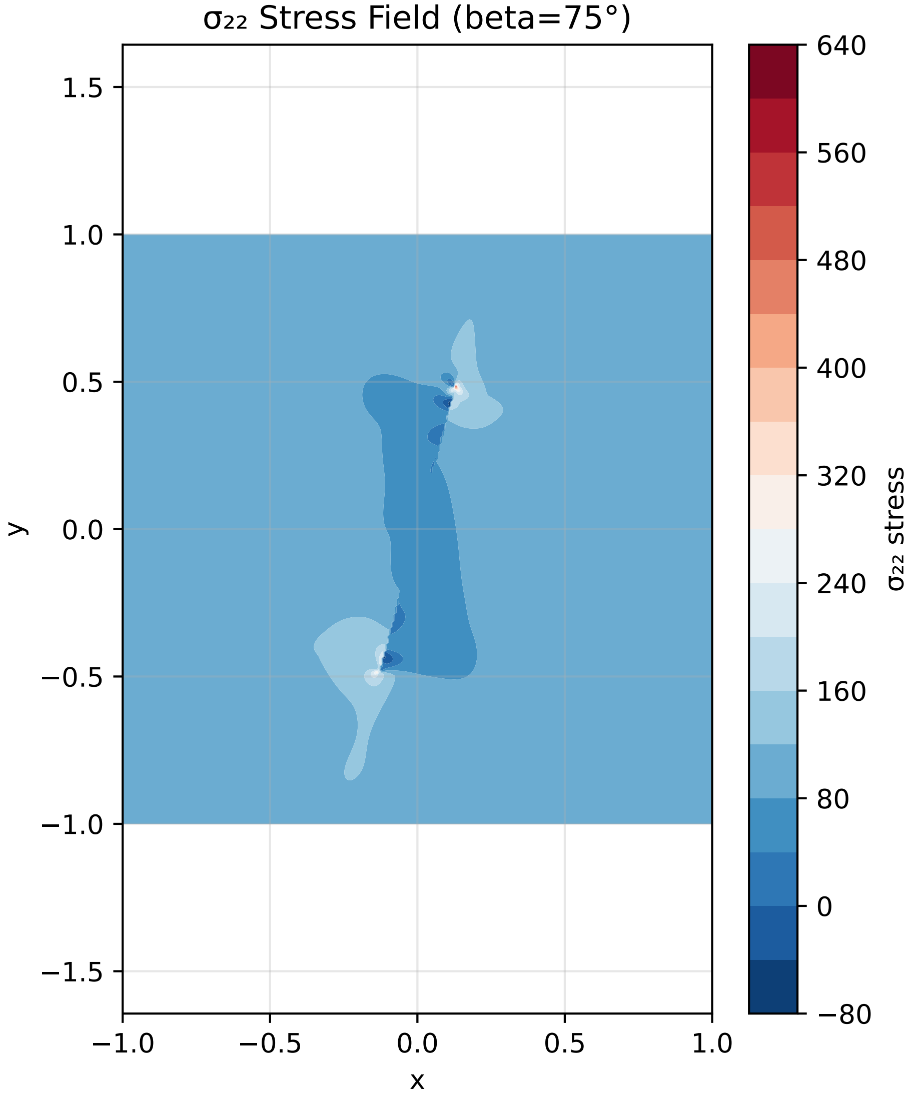
σ₂₂ Stress Field (beta=75°)
1.5
1.0
0.5
0.0
−0.5
−1.0
−1.5
y
−1.0
−0.5
0.0
0.5
1.0
x
640
560
480
400
320
240
160
80
0
−80
σ₂₂ stress
